# The Treatment of Parametritis by Dilating and Curetting the Uterus

**Published:** 1888-10

**Authors:** 


### Selections.

### THE TREATMENT OF PARAMETRITIS BY DILATING AND CURETTING THE UTERUS.

TRANSLATED PROM AN ARTICLE BY POULLET IN THE NOUVELLES ARCHIVES D'OBSTETRIQUE ET DE GYNECOLOGIE.

By VIRGIL O. HARDON, M. D.,

Professor of Obstetrics and Diseases of Women and Children, Atlanta Medical College, Atlanta, Georgia.

As a result of recent theories in regard to the pathology of uterine and peri-uterine inflammations—theories supported by Doléris in France, and by Walton and Hubert in Belgium—a great change is taking place in the therapeutics of these affections. After having been treated for a long time by methods exclusively medical—methods slow and uncertain in their results—inflammations of the uterus have now passed into the domain of surgery. They are to-day treated by measures which are rapid, safe and efficacious. Intra-uterine treatment by the curette has become a recognized method of procedure in cases of endometritis since one surgeon, Schroeder, has employed it in thousands of cases. In parenchymatous metritis this treatment, as introduced by Doléris, is much less generally accepted. I have, however, employed it extensively with great success.

But in cases of metritis accompanied by parametritis, all writers, even Doléris, himself, advise against this treatment. Courty, Churchill, Leblond, Thomas, Sinety, Emmet, Vesser, Doléris, Schroeder and Wathen, in fact, all who have written upon the subject, unanimously oppose dilatation of the uterus as long as the peri-uterine tissues are not free from inflammation. The application of this principle diminishes very materially the number of cases to which treatment is applicable, for even when peri-uterine inflammation cannot be positively demonstrated, the fear of its existence gives rise to hesitancy in operating.

I am aware of the objections unanimously offered by the best authorities against such procedure in cases of parametritis. I had never seen any opinions to the contrary when, in June, 1887, I was led by a series of observations, too extensive to be cited here, to treat parametritis actively by dilating and curetting the uterus.

Up to the present time I have curetted the uterus in twelve women who had peri-uterine inflammations of various degrees of acuteness, the larger part, however, having reached almost a chronic stage of the disease. But there was one who was in the acute stage of a recent parametritis. She had a temperature of 101.5, and walked bent over and with great pain. I dilated the uterus in three days by means of iodoformized sponge tents and curetted, repeating the treatment several times at short intervals, and in two months the inflammatory deposits had entirely disappeared.

These twelve patients were cured without any serious rise of temperature and much more rapidly than they could have been by the means hitherto in vogue. In eight of these patients the uterus was held in a position of retroflexion by adhesions resulting from old attacks of inflammation, some dating as far back as five or six years. I took advantage of the anæsthesia for the use of the curette to introduce into the uterus the largest of Budin's intra-uterine sounds, and, using it as a lever, I replaced the uterus so that the fundus rested against the abdominal wall and the pubes. In some of the cases the fixation of the retroflected uterus was such that I was unable to replace it at the first operation and was obliged to wait until the following month to complete the replacement.

Some of these retroflexions were so marked that the introduction of a uterine sound was almost impossible. In these cases I

sought to find some way in which I could obtain a flexible laminaria tent. I finally accomplished this object in the following manner: A large laminaria tent was first thoroughly boiled in a solution of corrosive sublimate. A small laminaria tent of such size that it would, if flexible, pass through the cervical canal, was passed through the opening in the centre of the large tent and allowed to remain until rendered flexible by imbibition of moisture. This takes from an hour to an hour and a half. When withdrawn the small tent is found to be rendered flexible without any increase of its size.

In the last ten months I have used the curette and the *ecouvillon* in fifty-one cases, including classic cases of endometritis, following rigidly the *technique* of Doléris. In all these cases the results have been marvelous. I am to-day thoroughly convinced of the safety of this treatment. Whatever may be the condition of the peri-uterine tissues as regards inflammation, vigorous antisepsis by creosote, iodoform or corrosive sublimate secures entire immunity from all complications. Accidents do not result from traumatism of the uterus of any sort; they result from infection through open blood vessels.

To show how completely I have lost my respect for the uterus, I will close by briefly reporting a recent case:

On the fourth of December, 1887, I was called by Drs. Urdy and Chalvel to see a case of pelvic peritonitis following a miscarriage at three months, which had taken place twenty-five days before. For fifteen days there had been incessant vomiting with entire inability to retain food, even ice being rejected. There was tympanites and moderate fever. The pulse was 100. The temperature had never exceeded 102.2, but there had been chills at various times, especially the evening before. The attending physicians, through fear of increasing the localized peritonitis, had made no examination. The local condition was therefore entirely unknown.

Yet I was not in the presence of timorous medical men. One was the surgeon to a large hospital, while the other had been for eight years an interne in Paris in the service of Péan. I was face to face with a doctrine which to-day I heartily oppose, *the*

*fear of traumatism of the uterus.* Under anæsthesia a complete examination, even with the uterine sound, showed metritis with septic material in the uterine cavity, and behind the uterus in the posterior cul-de-sac, an abscess of the size of a large turkey's egg. It would have been sufficient to open and wash out the cavity if this abscess had not the decomposing material within the uterus threatened to give rise to new centres of inflammation, as often happens.

I rapidly dilated the cervix, to the extent of half an inch, with Hegar's bougies. I cleared out the cavity of the uterus with the curette and the *ecouvillon*, and it was only when I had completely disinfected the womb that I proceeded to open the abscess. The following day food was retained, and in a month the patient had so far recovered as to be able to walk about the city. And yet we had replaced the uterus, dilated the cervix and treated the cavity with the curette and the *ecouvillon*, with a large abscess in the immediate neighborhood.

The attending physicians, astonished at what seemed to them like heroic practice, are to-day convinced that traumatisms, if made with proper precautions, are harmless even to the uterus. There as elsewhere there is no danger in operating with the wonderful aid furnished by antisepsis. The uterus is as tolerant as other tissues, but it absorbs more readily than any other the least particle of septic material.

